# Cytoreductive Surgical Treatment of Pleural Mesothelioma in a Porcine Model Using Magnetic-Resonance-Guided Focused Ultrasound Surgery (MRgFUS) and Radiofrequency Ablation (RFA)

**DOI:** 10.3390/tomography8050187

**Published:** 2022-09-03

**Authors:** Marcia Costa, Carolina Fernandes, Matt Eames, Arik Hananel, John P. Mugler, Jhosep Huaromo, Jack B. Yang, Jaime Mata

**Affiliations:** 1 Faculdade de Ciencias, Universidade de Lisboa, 1749-016 Lisboa, Portugal; 2 Focused Ultrasound Foundation, Charlottesville, VA 22903, USA; 3 Department of Radiology & Medical Imaging, University of Virginia, Charlottesville, VA 22903, USA

**Keywords:** high-intensity focused ultrasound, radiofrequency ablation, magnetic resonance imaging, mesothelioma

## Abstract

A combination of surgery and chemotherapy is the most effective treatment available for Malignant Pleural Mesothelioma (MPM). However, both cause significant collateral damage and cannot eliminate residual microscopic disease. This investigation aimed to compare and determine the feasibility of utilizing Radiofrequency Ablation (RFA) and Magnetic-Resonance-guided Focused Ultrasound Surgery (MRgFUS) as alternative treatments for MPM. A large animal tumor model was developed in 13 Yorkshire female pigs using the MSTO211H cell line. Two pigs were initially used to determine the cyclosporine dose required for immunosuppression and tumor development. Subsequently, 11 other pigs underwent tumor development. Of these 11, 2 died during cell inoculation. Small tumor masses and adhesions were present in the other 9, indicating mesothelioma development. Five pigs then received RFA treatment, and 4 pigs received MRgFUS treatment. Tumor model development and effect of the two treatments were examined using MRI and by necropsy. RFA and MRgFUS both successfully ablated approximately the same sized area in the same treatment time. This study demonstrates that RFA and MRgFUS are feasible for tumor debulking, and while MRgFUS requires more pretreatment planning compared to RFA, MRgFUS is a completely noninvasive procedure.

## 1. Introduction

Malignant Pleural Mesothelioma (MPM) is an aggressive type of cancer that develops from mesothelial cells lining the pleura. Exposure to asbestos fibers from industrial and environmental sources is the main cause of MPM [1,2]. A combination of surgery, such as pleurectomy/decortication (P/D) or extrapleural pneumonectomy (EPP), and chemotherapy is currently the most effective treatment available for MPM. Both treatment types are extremely aggressive and cause significant collateral damage to the body. They also lack the ability to eliminate residual microscopic disease, which can lead to the recurrence of the tumor [3,4,5]. As a result, the prognosis of MPM is very poor, with a median survival of 9–12 months [6].

Alternative treatments currently under investigation include Radiofrequency Ablation (RFA), a minimally invasive tumor treatment, and Magnetic-Resonance-guided Focused Ultrasound Surgery (MRgFUS), a completely noninvasive tumor treatment. RFA involves a thermal energy delivery system that emits an alternating current with high frequencies through an electrode needle. Ion agitation occurs in the tissue close to the needle because of this alternating current. Friction then turns the ion agitation in the tissue into heat, which can reach cytotoxic temperatures capable of denaturing proteins, melting lipid bilayers, and causing coagulation necrosis of nearby tumor cells [7]. While RFA is not MRI compatible, fluoroscopy and CT are imaging modalities that can be used for guidance.

High-Intensity Focused Ultrasound (HIFU) provides a beam capable of passing through skin and tissues to ablate a deep target area, such as a tumor. Absorption of the ultrasound energy by the tissue can lead to a rise in temperature greater than 60 °C, which can cause rapid cell death if this energy is maintained for longer than one second. Additionally, this technique creates a very sharp boundary between dead and live cells after ablation, minimizing damage to tissues outside of the target area. Use of MRI or ultrasound imaging as imaging modalities for HIFU guidance is another major component of this treatment. Because MRI provides high anatomical resolution, it allows for the accurate planning of the target area to be treated. Furthermore, it provides an anatomical image of the area within which temperature can be quantified, which cannot be achieved by ultrasound imaging [8].

Both RFA and HIFU are treatments that have been previously used in clinical practice to safely treat cancers and have the potential to be more effective and overcome many of the drawbacks found in surgery and chemotherapy when treating MPM [9,10]. As percutaneous image-guided ablation of tissue provides high local control rates of tumors with few complications, and has substantial efficacy for increasing patient survival, RFA and HIFU are compelling new possibilities for treatment of localized tumors [11,12]. Accordingly, the purpose of this investigation was to determine the feasibility of utilizing RFA and HIFU for the treatment of MPM.

## 2. Materials and Methods

Our protocol was approved by the University of Virginia’s Institutional Animal Care and Use Committee (IACUC-protocol code 3880). For this experiment, a total of 13 female Yorkshire pigs (~11 kg) were studied. A mesothelioma tumor model was initially developed to create a treatment prototype. This was done utilizing cyclosporine and a human mesothelioma cell line, MSTO211, which was purchased from the American Type Cell Culture (ACTC).

MSTO211 cell cultures were grown at 37 °C in a T-225 flask (Corning) using RPMI media (Invitrogen) and 10% FBS at 37 °C. Once confluence reached 70%, the cell medium was removed and the cells were rinsed with 0.25% (w/v) Trypsin—0.53 mM EDTA (Invitrogen). Trypsin-EDTA (10 mL) was added to each flask and cells were held under an inverted microscope until the cell layer was dispersed. Next, 10 mL of complete growth hormone was added to each flask and dispersed using pipette. Cells were then harvested and counted utilizing a hemocytometer and suspended in PBS at 10^6^ cells per mL

**Figure 1:**
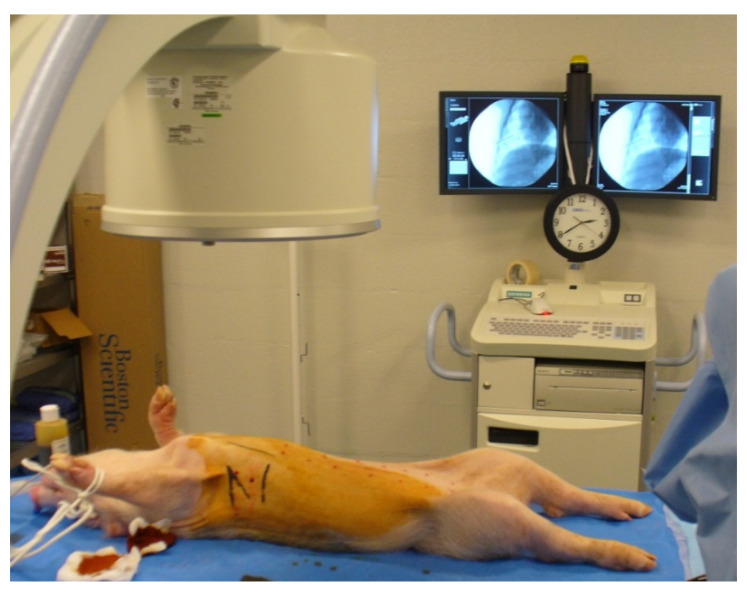
Setup environment for cell inoculation. Fluoroscopy allowed for the visualization of the heart and the ribs, guiding the procedure. Inoculations were performed on the right side in the intercostal spaces.

Two control pigs were initially utilized to determine the cyclosporine dose required for tumor model development. They were administered 10 mg/kg/day oral cyclosporine for immunosuppression, starting 7 days prior to the inoculation date. Pigs were anesthetized for the inoculation procedure (induction with 6 mg/kg Telazol and 2 mg/kg Xylazine, maintenance with 2% isoflurane), and the MSTO211 cells were injected into the right lower hemithorax, between the ribs, under fluoroscopy guidance (Siemens, Arcadis, PA) (Figure 1). Cell volume injected was dependent upon the number of cells grown in each colony. Cyclosporine was maintained for 4–12 weeks post-inoculation. The number of cells injected per pig as well as the cyclosporine dose is summarized in Table 1.

**Figure 2:**
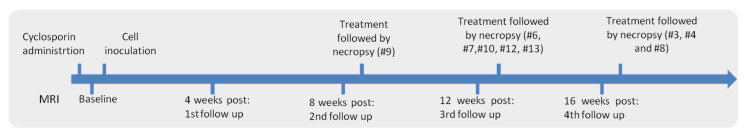
Timeline of the procedures.

For treatment pigs (#3–#13), the cyclosporine dose was increased to 20 mg/kg/day to increase the rate of tumor progression. Procedures followed to generate the tumor model were similar to those described above, with the number of cells and cyclosporine dose summarized in Table 1. All treatment animals were imaged at baseline and followed up every four weeks (Figure 2) using a 1.5T MR clinical scanner (Avanto, Siemens Healthcare, Malvern, PA, USA) with a body array and spine array coil simultaneously (Siemens, Malvern, PA, USA).

During follow-up imaging, the animals received one injection of contrast agent based on their weight in a 2.2 mL/kg dose (Gadolinium: Omniscan, Novaplus, PA or Magnevist, Bayer, NJ). Additionally, pigs were held underneath a ventilator-induced artificial breath hold to reduce motion artifacts. Five 2D MR pulse sequences were used during the follow ups to examine progression of the mesothelioma model: Haste (fast spin-echo T2-weighted sequence), Blade (T2-weighted sequence), VIBE (volume interpolated gradient echo sequence), True FISP (fast imaging with steady state precession), and TWIST (Time-resolved angiography sequence). After imaging, changes in the pig’s pleura were quantified utilizing True FISP images. True FISP was found to have the best sensitivity to fluid and tissue thickening, while VIBE was found to have good sensitivity to the development of adhesions. Image J was utilized for the manual selection of the areas of interest and calculations of the area and width. The *p* values were calculated utilizing t-test analysis.

Following development of the tumor model, animals were then treated with either percutaneous RFA or subcutaneous MRgFUS.

Five pigs were treated with RFA, utilizing an RF 3000 Radio Frequency Generator (Boston Scientific, Malborough, MA, USA) with an impedance-based feedback system and 200 Watts (W) of power capacity. The generator allowed for the connection of four electrosurgical ground pads for head dispersion (Electrosurgical Ground Pad with Safety Ring, Novaplus, Northfield, Illinois, USA). A 2.0 cm diameter LeVeen needle (Boston Scientific, Malborough, MA, USA) with an “umbrella” configuration was used for the ablations. The wattage was started at 30 W and then increased at 10 W/min until it reached a power of 60 W.

During RFA ablation, the pigs were anesthetized and kept under artificial ventilation in a supine position for the duration of the procedure. The area to be ablated was defined using prior MR images. RFA ablation was guided underneath fluoroscopy imaging (Siemens, Arcadis, Malvern, PA, USA), as the machine is not compatible with MRI. Distances between the heart, diaphragm, and chest wall were used to target the RFA probe. The probe was attached to a single position on the lung as it was being ventilated, thus allowing for the ablation to be smaller and more targeted. The endpoint for each ablation was determined by preset parameters, which enabled the equipment to be turned off automatically. Treatment took 30 min, and pigs were re-imaged 30–90 min following treatment with a 1.5 T MR scanner.

Four pigs were treated with MRgFUS at the University of Virginia’s Focused Ultrasound Center. The center utilizes an ExAblate 2000 OR system (Insightec, Haifa, Israel), with a portable patient table that is docked to a 3T MR scanner (General Electric, Chicago, Illinois, USA). Prior to the procedure, Insightec software was used for three-dimensional planning to reduce treatment times and to increase treatment accuracy. During the procedure, Insightec software was used for MR thermometry. An escalation study was conducted to determine the power required for successful ablation of the lung pleura.

During the procedure, animals were anesthetized and held in the lateral position. Planning of the localization of the focal spot was done with a low-level ablation. Following the low-level ablation, coordinates of the focal spot were repositioned, and the power and duration of sonication adjusted. During treatment, MRI images were obtained utilizing LAVA and FIESTA pulse sequences, which are equivalent to VIBE and True FISP sequences in the Siemens MR scanners. Total treatment time, including MRI imaging, was approximately 15 min. The pigs were re-imaged with 3T MRI following MRgFUS treatment.

After re-imaging, animals in both treatment groups were immediately euthanized (Euthasol, 1 mL/4.5 kg). An incision was made parallel to the sternum, the ribs were cut laterally, and the thoracic cavity was exposed anteriorly. The thoracic cavity was examined for development of the mesothelioma tumor model, as well as for any subcutaneous injuries that may have been caused by RFA/MRgFUS. The skin, muscle, pericardium, and lung pleura were also examined for any other collateral damage that might have occurred.

## 3. Results

### 3.1. Tumor Model

To determine the relationship between cell numbers and the rate of tumor development, the two control pigs received an unequal number of mesothelioma cells. Analysis of both MRI and necropsy revealed that pig #2 had faster disease development, having received more than twice the number of cells when compared to pig #1 (13 mL vs. 6 mL). However, neither pig showed tumor masses, so the cyclosporine dose was doubled for the treatment pigs. Mild side effects, such as vomiting and diarrhea, were observed after increasing the dose, but these were not debilitating so the dosage was not reduced.

Pigs #5 and #11 died immediately after the cell inoculation procedure. Based on MR images, pig #5 developed mild inflammation in the left lung, and, following inoculation, the heart stopped beating. Pig #13 was inoculated as a replacement for pig #5. Pig #11 was unable to continue breathing upon ventilator withdrawal following inoculation, and no pig replaced pig #11.

**Figure 3:**
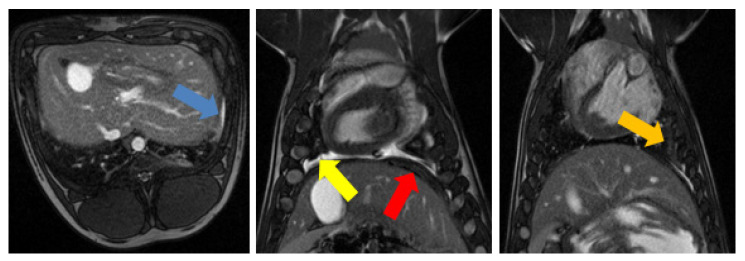
Main mesothelioma findings in MRI. (**Left**): True fast imaging with steady state precession (FISP) transverse image of pig #8 showing pleural thickening (blue arrow); (**Middle**): True FISP coronal image of pig #8 showing pleural fluid (yellow arrow) and the increased thickness of diaphragm (red arrow); (**Right**): True FISP coronal image of pig #8 showing adhesions (orange arrow).

The most prominent sign of the mesothelioma model on MRI was the presence of pleural effusion, which was seen at several time points in all pigs. Pleural effusion was characterized by a hyperintense region that was visible in the True FISP images (Figure 3). Furthermore, increases in pleural, diaphragm, and pericardium thickness were observed for all animals (Table 2). A slight increase in the lung tissue was also observed in several pigs, which may have been related to transient tissue inflammation.

**Figure 4:**
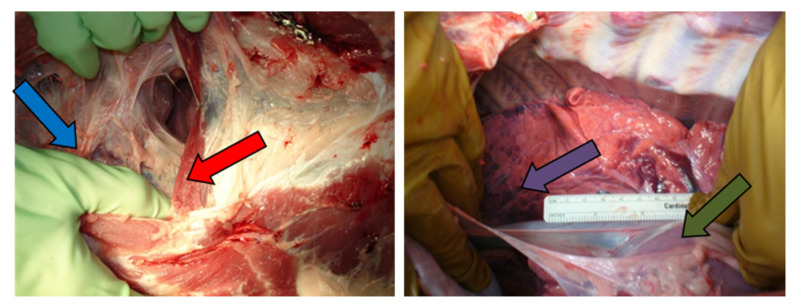
Macroscopic findings during necropsy: adhesions (blue arrow), increased diaphragm thickness (red arrow), abnormal color in the lungs (purple arrow), and increased pericardium thickness (green arrow).

Several signs of mesothelioma development were prominent during necropsy. In particular, 81% of the pigs developed adhesions following mesothelioma cell inoculation (Figure 4). Pigs #1 and #4 had no sign of adhesion development, while Pig #8 had the most adhesion development, making it difficult to access the thoracic cage during necropsy. In seven of the pigs, the lung was hyperpigmented and lacked normal consistency, which may have been indicative of prenecrotic tissue.

### 3.2. Radiofrequency Ablation

**Figure 5:**
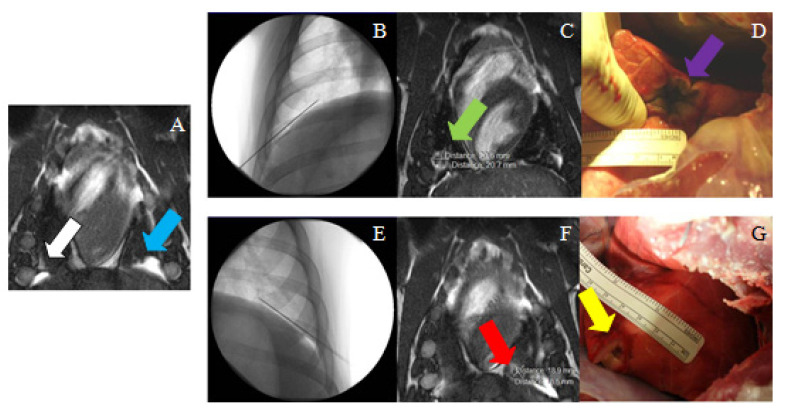
(**A**) MR image (True FISP) used for treatment planning in the right (white arrow) and the left (blue arrow) sides for pig #4. (**B**) Needle placement in the right side (fluoroscopy image); (**C**) post-ablation MRI (True FISP), showing the ablated area in the right side (green arrow); (**D**) necropsy image showing the ablated area in the right diaphragm (purple arrow). (**E**) needle placement in the left side (fluoroscopy image); (**F**) post-ablation MRI (True FISP), showing the ablated area in the left side (red arrow); (**G**) necropsy image showing the ablated area in the left diaphragm (yellow arrow).

The results of radiofrequency ablation treatment revealed that it was possible to obtain ablations more than 2 cm in approximately 30 min of treatment. Figure 5 shows the results of the pleural RFA treatments of pig #4, which generated a 1.9 cm diameter ablation on the left side of the pleural space, and a 2 cm diameter ablation on the right side of the pleural space.

### 3.3. Magnetic-Resonance-Guided Focused Ultrasound

**Figure 6:**
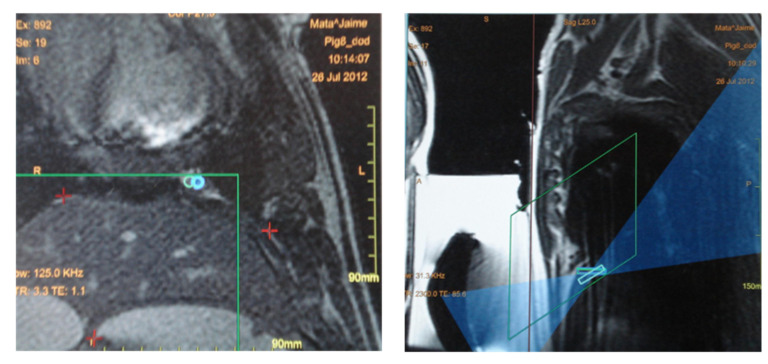
(**Left**) The blue circles in this coronal MRI above the diaphragm represent two focal spots planned, both localized inside the green square which represents the area available for treatment. The red crosses in the middle of the image mark the position of the diaphragm. (**Right**) Sagittal view of the image used for treatment planning. The rectangles represent the depth of the focal spots. The blue region corresponds to the beam path across the adjacent tissue.

The first animal to be treated with MRgFUS was pig #8. As a result of the escalation study, pig #8 received the lowest amount of acoustic energy in seven focal spots. The first five were targeted direct hits and the beam was not angulated as the focal points were between the ribs. For the last two focal spots targeted, the beam was angulated 32° to treat the area closer to the bottom of the last rib (Figure 6). Ablations were conducted at approximately 200 W for 20 s, for an energy output of 4000 J. The ablations were visible on MR subtraction images, with a 1.5 by 1.3 cm^2^ ablation in the lower right lung. Upon necropsy, the corresponding ablation spot was superficial and elongated, much larger than the perceived image on MRI.

Pig #10 was the next pig to be treated with MRgFUS. As the mesothelioma tumor model was not as developed in this pig, there were only three focal spots, which were each ablated twice. Each focal spot had the same power delivered at 326 W for 12 s, for an energy output of 3912 J. The beam was conducted using an intercostal method to avoid absorption by the ribs and was angulated between 7° and 21°. MRI analysis revealed an ablated area of approximately 1.4 by 1.4 cm^2^, near the heart, in the lower lobe of the left lung. Upon necropsy, there was a small and localized ablation (<1 cm) in the anterior part of the left lung.

**Figure 7:**
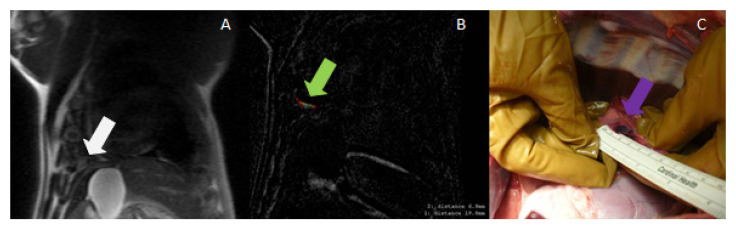
(**A**) MRI (localizer) used for treatment planning in the pleura (white arrow); (**B**) Post-ablation MRI (subtraction of LAVA pulse sequence images before and after ablation), showing a very small ablated area near the diaphragm (green arrow); (**C**) necropsy image showing the ablated area in the pleura (purple arrow).

For pig #12, the time of sonication was increased from a 12 s duration to 21 s at 328 W, increasing the energy output to 6888 J. Each focal spot was ablated twice with a beam angle of 32°. Consequently, the ablation created was larger, with MRI showing a 2.4 by 1.6 cm^2^ ablation in the lower lobe of the right lung. Upon necropsy, analysis revealed an elongated lesion that measured 3 by 1.5 cm^2^ in the lower lobe of the right lung (Figure 7).

Pig #13 was the final pig treated with MRgFUS and received the highest amount of acoustic energy. Pig #13 had seven focal spots and each focal spot was sonicated twice, with each sonication at approximately 328 W for 20 s, for an energy output of 6560 J. As the ablation area was very close to the diaphragm, errors due to motion were expected. In addition, during the ablation of the 4th focal spot, some reflection from the ribs occurred and the power was reduced from 328 W (maximum) to 300 W. The beam was angulated between 24° and 30°. On MRI, the ablation was visible as a hyperintense region that was 4.2 × 2.5 cm^2^ on the right side of the diaphragm. Upon necropsy, ablation of the diaphragm was observed, as well as an ablation area in the middle lobe of the lung that was consistent with MRI analysis. 

## 4. Discussion

The results of this study demonstrate the ability to generate an in vivo porcine mesothelioma model and perform ablations of lung pleura utilizing percutaneous RFA and subcutaneous MRgFUS. The tumor model was characterized by the presence of thickened diaphragm, adhesions, and consistent pleural effusion.

Within the lung pleura, large tumor masses were not observable. This may have been due to the delivery technique performed, which involved suspending the cells within PBS and then injecting this mixture into the right lung hemithorax. As a result, there was diffuse thickening of the diaphragm and pericardium within the thoracic cavity rather than focal, localized tumor masses. It may be possible to increase the concentration of tumor cells by using a Matrigel substance in order to localize development of mesothelioma cells. In addition, an increased post-inoculation period may further increase the likelihood of larger tumor masses that are more characteristic of mesothelioma. However, as histopathological analysis of the thickened pericardium and diaphragm was not conducted, we cannot definitively conclude that a pure mesothelioma model was created.

Prior to beginning the immunosuppression treatment, the dosage of cyclosporine was calculated based upon the dosage that humans receive. However, it has been reported that the blood concentration of cyclosporine within pigs is lower than that of humans with the same dosage both orally and IV, and higher dosages of cyclosporine are required as a result [13,14]. In addition, one of the difficulties with this study was an interruption in the supply of cyclosporine. Due to supply constraints, we were unable to immunosuppress the animals for longer than 12 weeks and had to stop cyclosporine treatment prior to 12 weeks for some animals. Despite this, animals showed no signs of regression in tumor growth, and animals treated with cyclosporine for only 4 weeks still showed tumor development. Consequently, we concluded that cyclosporine administration is needed to induce the MPM, but constant administration may not be required after an initial period.

Due to production constraints, the supplier of the mesothelioma cells provided a set volume of cells per shipment. Depending on the shipment, different volumes of cells were injected into the hemithorax of the animals. There appeared to be a relationship between the number of cells injected and the rate of progression, as the two control pigs had different levels of disease severity. However, past a certain point, the number of cells injected did not appear to correlate to the severity or progression of disease within the treatment pigs.

Other issues involved the measurement of the thickness of the diaphragm and pericardium. The pericardium in pigs generally increases with heart size and has an average thickness of 0.2 mm [15,16,17]. It is possible that the pericardial fluid was inadequately measured with the pericardium itself, resulting in higher thickness values. It is unlikely that the change in pericardium thickness was caused purely by the disease model. On the other hand, the diaphragm thickness increased in the first 8 weeks after inoculation before reaching a plateau. This could be due to the disease model as the adhesions in the thoracic cavity restricted movement of the lungs, resulting in an increased workload on the diaphragm to maintain normal respirations. The two animals that died after the inoculation procedure did not show any signs of disease or bleeding, so their death was concluded to be from cardiac arrest of unknown etiology. Fluoroscopy was performed, but no necropsy was performed due to the high risk of exposure from the recently delivered mesothelioma cells.

Initially, the objective of our study was to utilize percutaneous RFA ablation to treat tumor masses in the pleura. However, due to the lack of large tumor masses and the consistent presence of adhesions, target objectives were changed to breaking the adhesions instead. One advantage found with the use of the RFA needle was the increased accuracy due to the physical contact between the electrode and the lung pleura, which allowed the probe to move with the lungs during ventilation. However, one disadvantage was that the RFA probe was not compatible with MRI imaging, and treatment guidance was performed using fluoroscopy imaging. Targeting was performed as close as possible to MRI-observed adhesions and structural features, but it was not always possible to precisely target the area of interest.

Another disadvantage was the manufacturer roll-off phase, which would shut down the treatment if a rapid decrease in temperature was detected. If a roll-off occurred, then the system would be restarted at half of the roll-off power, 30 s later. When performing the experiment, the left side of the animals tended to roll-off quicker than the right side, thus delivering less power for ablation. Consequently, the right side had larger ablations, and may have resulted in overablation. We theorize that the difference in the roll-off time between the two sides may have been due to different tissue/tumor properties, as the right side had higher amounts of tumor mass.

MRgFUS has issues with ablation of lung pleura due to the presence of air and a high density of air–tissue interfaces. Our study attempted to navigate this issue by treating the lung surface pleura only. As a result of MR imaging, we were able to obtain ablations that were both visible on MRI and accurate when observed on necropsy. These lesions were similar in size to RFA, and both treatments took approximately the same amount of time to conduct. In addition, as these ablations were conducted during an artificial breath-hold, lung movement was reduced and increased the accuracy of the procedure.

For this pilot study, the acoustic power and delivery time were progressively increased to evaluate the size, depth, and side effects of the high-power sonications. These power escalations showed that pleural effusions were not side effects of the higher acoustic power or treatment time of MRgFUS and helped to set a power base for future studies to utilize. To avoid possible skin burns due to higher amounts of acoustic energy, future studies may utilize acoustic reflector materials, such as foam or cork placed in a near field underneath a gel phantom to shield the skin from the ultrasound beam. Preliminary results have shown that it is possible to avoid skin burns through the insertion of these materials, which increases the depth of sonications and decreases the radius of the reflector [18,19]. Furthermore, recent studies have demonstrated that one-lung flooding is a viable and safe approach, and the use of a liquid medium may be another possible approach for the use of MRgFUS within the lung pleura [20].

Some abnormalities noted in the use of MRgFUS included unusually high heat readings by the MR thermometry software. Other studies have reported discrepancies in MR thermometry as well, noting a difference between applied in vivo temperatures and software-read temperatures [21]. Improvements in thermometry software would increase the accuracy of MRgFUS treatment, as well as decreasing the possibility of collateral damage. Other improvements could be made by adjusting the transducers used—for this experiment, we used an ExAblate 2000 transducer, which is utilized for uterine fibroids. With the development of new transducers for the treatment of hepatocellular carcinoma and breast cancer, there may be possible alternative transducers for this application.

Overall, we were able to create a porcine mesothelioma model and to successfully ablate the lung pleura utilizing both RFA and MRgFUS. When compared to RFA, MRgFUS allowed us to define the shape (via different beam angulations) of the lesion for consistent and accurate treatments. Although MRgFUS requires longer pretreatment planning, the ablation area could be verified prior to the treatment, enabling the operator to ensure the sonication was performed only in the targeted areas.

## 5. Conclusions

Since this is a new area of research, this project allowed us to study some of the parameters of MRgFUS required to obtain a successful ablation of the pleura. We were able to prove the feasibility of both techniques (MRgFUS and RFA) and obtained ablation areas with approximately similar sizes. Nevertheless, this topic requires more research and another study with a larger number of animals will be necessary to obtain statistical significance of the results. MRgFUS has many advantages as it does not require incisions, thus lowering the probability of infection, encouraging faster recoveries, and creating a potentially less expensive procedure. MRgFUS offers a new treatment modality that can be tested with future studies and validated through clinical trials.

## Figures and Tables

**Table 1:** Summary for volumes of the MSTO-211H solution injected, dose of cyclosporine administered, and type of treatment received by each pig.

Animal #	Cell Solution Injected (mL)	Cyclosporine Dose (mg/kg/day)	Type of Treatment Received
1	6.0	10 (until euthanasia)	Test (none)
2	13.0	10 (until euthanasia)	Test (none)
3	11.0	20 (until 12 weeks post-inoculation)	RFA
4	5.0	20 (until 12 weeks post-inoculation)	RFA
5	5.5	20 (died after inoculation)	--
6	9.0	20 (until 12 weeks post-inoculation)	RFA
7	7.0	20 (until 8 weeks post-inoculation)	RFA
8	7.0	20 (until 8 weeks post-inoculation)	MRgFUS
9	10.0	20 (until 8 weeks post-inoculation)	RFA
10	5.0	20 (until 4 weeks post-inoculation)	MRgFUS
11	6.0	20 (died after inoculation)	--
12	8.0	20 (until 4 weeks post-inoculation)	MRgFUS
13	8.0	20 (until 4 weeks post-inoculation)	MRgFUS

**Table 2:** Change in pericardium and diaphragm thickness throughout follow up.

Pericardium Thickness (mm)	Pig #3	Pig #4	Pig #6	Pig #7	Pig #8	Pig #9	Pig #10	Pig #13	Average	*p*-Value
Baseline	3.17	3.00	2.54	2.88	3.00	3.90	3.35	3.50	3.17	-
4 weeks	3.24	3.93	2.34	3.14	3.65	6.29	5.73	5.19	4.19	0.0255
8 weeks	3.20	5.11	5.51	4.76	4.80	7.42	6.20	6.21	5.40	0.0006
12 weeks	5.17	5.00	7.02	5.01	8.98	-	-	-	6.24	0.0153
16 weeks	6.21	6.06	-	-	-	-	-	-	6.14 *	- *
Baseline-final % change	96%	102%	176%	74%	200%	90%	85%	77%	94%	-
**Diaphragm thickness (mm)**										
Baseline	2.84	3.01	5.30	5.02	4.27	4.12	3.40	5.01	4.12	-
4 weeks	6.00	4.00	6.13	5.89	7.43	5.72	5.04	5.93	5.77	0.0021
8 weeks	7.67	5.21	6.10	6.74	8.55	7.47	5.33	6.48	6.69	0.0014
12 weeks	7.06	4.80	7.57	7.48	9.62	-	-	-	7.31	0.0087
16 weeks	9.52	5.85	-	-	-	-	-	-	7.68 *	- *
Baseline-final % change	236%	94%	43%	49%	125%	81%	57%	29%	86%	-

* The average listed is for two pigs only. Normal distribution did not hold, so no *p*-value was obtained.

## Data Availability

Not applicable.
